# *α*7 Nicotinic Receptor Promotes the Neuroprotective Functions of Astrocytes against Oxaliplatin Neurotoxicity

**DOI:** 10.1155/2015/396908

**Published:** 2015-06-03

**Authors:** Lorenzo Di Cesare Mannelli, Barbara Tenci, Matteo Zanardelli, Paola Failli, Carla Ghelardini

**Affiliations:** Department of Neuroscience, Psychology, Drug Research and Child Health (NEUROFARBA), Pharmacology and Toxicology Section, University of Florence, 50139 Florence, Italy

## Abstract

Neuropathies are characterized by a complex response of the central nervous system to injuries. Glial cells are recruited to maintain neuronal homeostasis but dysregulated activation leads to pain signaling amplification and reduces the glial neuroprotective power. Recently, we highlighted the property of *α*7 nicotinic-acetylcholine-receptor (nAChR) agonists to relieve pain and induce neuroprotection simultaneously with a strong increase in astrocyte density. Aimed to study the role of *α*7 nAChR in the neuron-glia cross-talk, we treated primary rat neurons and astrocytes with the neurotoxic anticancer drug oxaliplatin evaluating the effect of the *α*7 nAChR agonist PNU-282987 (PNU). Oxaliplatin (1 *μ*M, 48 h) reduced cell viability and increased caspase-3 activity of neuron monocultures without damaging astrocytes. In cocultures, astrocytes were not able to protect neurons by oxaliplatin even if glial cell metabolism was stimulated (pyruvate increase). On the contrary, the coculture incubation with 10 *μ*M PNU improved neuron viability and inhibited apoptosis. In the absence of astrocytes, the protection disappeared. Furthermore, PNU promoted the release of the anti-inflammatory cytokine TGF-*β*1 and the expression of the glutamate-detoxifying enzyme glutamine synthetase. The *α*7 nAChR stimulation protects neurons from oxaliplatin toxicity through an astrocyte-mediated mechanism. *α*7 nAChR is suggested for recovering the homeostatic role of astrocytes.

## 1. Introduction

The development of painful neuropathies is a dose limiting side effect of commonly used chemotherapeutic agents, including platinum drugs [1–3]. Repeated oxaliplatin infusions induce grade ≥2 neuropathy in 40–50% of patients receiving standard treatment regimens, with grade ≥3 neuropathy in 10–20% of patients [4, 5]. After a cumulative dose of 750–850 mg/m^2^ 82–93% of patients experience symptoms of neuropathy including 12–34% with grade 3/4 neuropathy [6, 7].

The pathophysiological mechanisms of chemotherapy neurotoxicity are still little known. Several nervous cell alterations are involved in the final neuron damage [8], limiting the possibilities to develop effective therapies [9, 10]. In a rat model of oxaliplatin neuropathy, we have highlighted the relationship between the peripheral nervous injuries (with the resulting aberrant somatosensory processing) and the activation of glial cells (microglia and astrocytes) in both spinal cord and supraspinal areas [11]. Glial activation, in particular astrocytes, is strictly related to oxaliplatin-dependent pain since glial inhibitors reduce mechanical and thermal hypersensitivity [12]. On the other hand, glial cells evoke neuroprotective mechanisms [13] and the block of glial-related signals impairs functional recovery after nerve injuries [14], suggesting that* tout court* glial inhibition may relieve pain but hinders the rescue mechanisms that protect nervous tissue from the damage triggering chronic pain. Recently, the stimulation of the *α*7 subtype of the nicotinic receptors (*α*7 nAChR; see [15, 16] for review) has emerged as effective in reducing oxaliplatin-dependent pain and preventing damage to the nervous tissue. These effects parallel with increased glial cell number in a region-specific manner [17]. *α*7 nAChR seems to be able to modulate glial cells distinguishing between the positive, protective component of microglia and astrocyte signaling and the pathological painful pathway. Nevertheless, the relevance of glial cells in the *α*7 nAChR signaling network as well as the molecular mechanisms remains unclear.

Aimed at improving the knowledge about the role of *α*7 nAChR in the neuron-glia signaling, primary neurons and astrocytes were cocultured in the presence of PNU-282987 (PNU), a selective and potent *α*7 nAChR agonist [18–20]. The protective component of *α*7 nAChR stimulation was focused using the anticancer drug oxaliplatin as neurotoxic agent. According to our research, oxaliplatin-induced alterations in nervous cell cultures represent a valuable* in vitro* model for studying cell damage mechanisms and screening novel protective molecules [21].

## 2. Materials and Methods

### 2.1. Cell Culture Preparation

Sprague-Dawley rats (Harlan, Varese, Italy) were used to obtain primary cell cultures. Animals were housed in CeSAL (Centro Stabulazione Animali da Laboratorio, University of Florence), in 26 × 41 cm cage, were fed with standard laboratory diet and tap water* ad libitum*, and were kept at 23 ± 1°C with a 12 h light/dark cycle, light at 7 a.m. All animal manipulations were carried out according to the European Community guidelines for animal care (DL 116/92, application of the European Communities Council Directive of November 24, 1986, 86/609/EEC). Formal approval to conduct the experiments described was obtained from the Animal Subjects Review Board of the University of Florence. The ethical policy of the University of Florence complies with the Guide for the Care and Use of Laboratory Animals of the US National Institutes of Health (NIH Publication number 85-23, revised 1996; University of Florence assurance number: A5278-01).

Primary cultures of astrocytes were obtained according to the method described by McCarthy and de Vellis [22]. Briefly, the cerebral cortex of newborn (P1–P3) Sprague-Dawley rats (Harlan, Padova, Italy) was dissociated in Hanks balanced salt solution containing 0.5% trypsin/0.2% EDTA and 1% DNase (Sigma-Aldrich, Milan, Italy) for 30 min at 37°C. The suspension was mechanically homogenized and filtered. Cells were plated in high-glucose DMEM with 20% FBS (Life Technologies, Milan, Italy). Confluent primary glial cultures were used to isolate astrocytes, removing microglia and oligodendrocytes by shaking. After 21 days of culture, astrocytes were plated according to experimental requirements. Experiments were performed 21 days after cell isolation. GFAP-positive cells were 95–98% [23].

Cortical neurons were isolated from rat embryos (embryonic days 14–16; Sprague-Dawley rats; Harlan, Padova, Italy). The cortex was dissociated in Hanks balanced salt solution containing 0.5% trypsin/0.2% EDTA and 1% DNase for 10 min at 37°C under mild stirring. The suspension was mechanically homogenized and filtered. Cells were plated in Neurobasal Medium supplemented with 2% B27 Supplement and 2 mM* L*-glutamine (Life Technologies, Milan, Italy) to allow the selective growth of neurons according to [24]. After 5 days of culture, neurons were plated according to experimental requirements.

Both astrocyte and neuron cultures were released from the culture plates by treatment with 0.5% trypsin in PBS containing 0.03% EDTA for 1 min and allowed to seed for at least 24 h before being used for the experiments.

### 2.2. Neuron Astrocyte Coculture

Neurons and astrocytes were separately cultured for 5 and 21 days, respectively. Then astrocytes were plated in transwells (0.45 *μ*M pore size; BD Biosciences, Durham, NC, USA) while neurons were plated in multiwells as described below. The day after plating, inserts containing astrocytes were placed on the wells containing primary neurons and, after 24 h, treated with oxaliplatin (Sequoia Research Products, Pangbourne, UK) in the presence or absence of 10 *μ*M PNU-282987 (PNU-282987; Sigma-Aldrich, Milan, Italy). In this modeling, although neurons and astrocytes face each other, they are separable, and the effect of soluble factors released from activated astrocytes on neurons can be studied, allowing separate analysis of neuronal and glial populations [25].

### 2.3. Cell Viability Assay

Cell viability was evaluated by the reduction of 3-(4,5-dimethylthiazol-2-yl)-2,5-diphenyltetrazolium bromide (MTT; Sigma-Aldrich, Milan, Italy) as an index of mitochondrial compartment functionality. For experiments in monocultures, primary neurons or astrocytes were plated in 96-well cell culture plates (10^4^ cells/well) and grown until confluent. For experiments in coculture primary neurons were plated in 24-well plates (2 10^5^ cells/well) while primary astrocytes were plated in appropriate transwells (8 10^4^ cells/well) and grown until confluence. Cells were treated for 48 h with oxaliplatin (0.3–100 *μ*M) in the presence or absence of PNU (10 *μ*M). After extensive washing, 1 mg/mL MTT was added to each well and incubated for 2 h at 37°C. After washing, formazan crystals were dissolved in 100 *μ*L dimethyl sulfoxide. The absorbance was measured at 580 nm. Experiments were performed in quadruplicate on at least three different cell batches.

### 2.4. Caspase-3 Activity

In monoculture experiments, neurons or astrocytes were plated in 6-well plates (5 10^5^ cells/well) and grown until confluence. In coculture experiments neurons were plated in 6-well plates (7 10^5^ cells/well) while astrocytes were plated in appropriate transwells (4.5 10^5^ cells/well). Incubation with increasing concentrations of oxaliplatin (0.3–100 *μ*M) was allowed for different times (4, 8, and 48 h) in the absence or presence of 10 *μ*M PNU. After treatment, cells were scraped in 100 *μ*L lysis buffer (200 mM Tris-HCl buffer, pH 7.5, containing 2 M NaCl, 20 mM EDTA, and 0.2% Triton X-100). Fifty *μ*L of the supernatant was incubated with 25 *μ*M fluorogenic peptide caspase substrate rhodamine 110 bis(N-CBZ-*L*-aspartyl-*L*-glutamyl-*L*-valyl-*L*-aspartic acid amide) (Molecular Probes, Milan, Italy) at 25°C for 30 min. The amount of cleaved substrate of each sample was measured in a 96-well plate fluorescence spectrometer (PerkinElmer; excitation at 496 nm and emission at 520 nm).

### 2.5. TGF-*β*1 Dosage

In monoculture experiments neurons or astrocytes were plated in a 6-well plate (5 10^5^ cells/well). In coculture experiments neurons were plated in 6-well cell culture plates (7 10^5^ cells/well), while astrocytes were plated in specific transwells (4.5 10^5^ cells/well). Cells were then treated with oxaliplatin 1 *μ*M for 48 h in the presence or absence of 10 *μ*M PNU. After treatment, the culture medium was collected and used for determining TGF-*β*1 by ELISA kit (BioLegend, Inc., USA). Culture medium samples were activated by acidification and processed according to the manufacturer protocol. The absorbance was measured at 450 nm. Declared assay sensibility is 1 pg/mL.

### 2.6. Pyruvate Assay

In order to evaluate astrocyte metabolism, pyruvate intracellular concentration was measured. The dosage is based on the conversion of pyruvate to acetyl phosphate, hydrogen peroxide, and carbon dioxide catalyzed by pyruvate oxidase. In the presence of horseradish peroxidase, H_2_O_2_ reacts stoichiometrically with 10-acetyl-3,7-dihydroxyphenoxazine to produce resorufin. Resorufin fluorescence is analyzed with an excitation wavelength between 530 and 540 nm and an emission wavelength between 585 and 595 nm.

Astrocytes were plated in a 6-well plate (5 10^5^ cells/well). After treatments (1 *μ*M oxaliplatin for 48 h in the presence or absence of 10 *μ*M PNU) cells were scraped. The cell pellet was treated with 0.5 mL 0.25 M metaphosphoric acid on ice for 5 min to deproteinate the sample. After centrifugation (10000 ×g for 5 min at 4°C), the pellet was neutralized by adding 25 *μ*L K_2_CO_3_ (5 M). After centrifugation, the supernatant was analyzed following the manufacturer's instruction (Cayman, Ann Arbor, MI, USA).

### 2.7. Western Blot Analysis

After incubation, astrocyte cell cultures were washed once with PBS and scraped on ice with lysis buffer containing 50 mM Tris-HCl pH 8.0, 150 mM NaCl, 1 mM EDTA, 0.5% Triton X-100, and Complete Protease Inhibitor (Roche, Milan, Italy). Suspensions were then collected, subjected to a freeze-thaw cycle, and centrifuged at 13,0006 g for 10 min at 4°C. Protein concentrations were quantified by bicinchoninic acid assay. Forty *μ*g of each sample was resolved with 10% SDS-PAGE before electrophoretic transfer onto nitrocellulose membranes (Bio-Rad, Milan, Italy). Membranes were blocked with 5% nonfat dry milk in PBS containing 0.1% Tween 20 (PBST) and then probed overnight at 4°C with primary antibody specific versus glutamine synthetase (1 : 2500; 45 kDa; Millipore, Billerica, MA, USA) or GAPDH (1 : 1000; 38 kDa; Cell Signaling, Boston, MA, USA). Membranes were then incubated for 1 hour in PBST containing the appropriate horseradish peroxidase-conjugated secondary anti-rabbit (1 : 5000; Cell Signaling, USA) or anti-mouse antibody (1 : 2000; Santa Cruz, USA). ECL (Enhanced Chemiluminescence Pierce, Rockford, IL, USA) was used to visualize the peroxidase-coated bands. Densitometric analysis was performed using the “ImageJ” analysis software (ImageJ, NIH, Bethesda, MD, USA) and results were normalized to GAPDH immunoreactivity as internal control. Values were reported as percentages in comparison to control which was arbitrarily fixed at 100%.

### 2.8. Statistical Analysis

Results were expressed as mean ± S.E.M. and analysis of variance (ANOVA) was performed. A Bonferroni significant difference procedure was used as post hoc comparison. All assessments were made by researchers blinded to cell treatments. Data were analyzed using the “Origin 8.1” software (OriginLab, Northampton, USA).

## 3. Results

**Figure 1:**
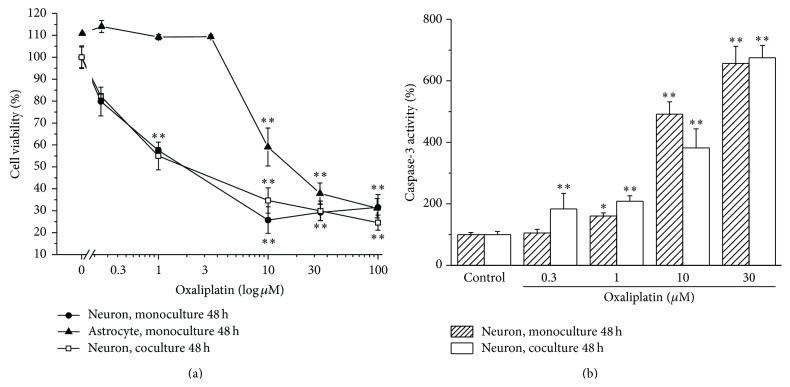
Oxaliplatin toxicity in mono- and cocultures. (a) Cell viability. Neuron monoculture (10^4^ cells/well), astrocyte monoculture (10^4^ cells/well), and neuron astrocyte coculture (2 10^5^ neurons/well and 8 10^4^ astrocytes/well) were incubated with oxaliplatin (0.3–100 *μ*M) for 48 h. Viability of neurons in coculture was quantified by MTT assay and compared with the viability of neurons or astrocytes in monoculture. Values are expressed in percentage of control absorbance (0 oxaliplatin) as mean ± S.E.M. of 6 experiments. Control condition absorbance was fixed to 100%. ^*∗∗*^ *P* < 0.01 versus control. (b) Caspase-3 activity. Neuron monoculture (7 10^5^ cells/well) or neuron astrocyte coculture (7 10^5^ neurons/well and 4.5 10^5^ astrocytes/well) was incubated with oxaliplatin (0.3–100 *μ*M) for 48 h. The enzymatic activity of neuronal component of the coculture was compared to that of neuron monocultures. The enzymatic activity was measured by a fluorescent assay. Values are expressed as percent of control caspase-3 activity arbitrarily set as 100%. Bars represent mean ± S.E.M. of 3 experiments. One-way ANOVA was performed followed by a Bonferroni significant difference procedure. ^*∗*^ *P* < 0.05 and ^*∗∗*^ *P* < 0.01 versus control.

Oxaliplatin induced a concentration-dependent decrease of neuron viability after 48 h incubation. As shown in Figure 1(a) (neuron, monoculture), significant effects were evoked by 1 *μ*M oxaliplatin (40% decrease) reaching a plateau for decreased viability at concentrations higher than 10 *μ*M (about 70% decrease). The same cell treatment increased the activity of caspase-3 in a concentration-dependent way: 1 *μ*M oxaliplatin enhanced the enzymatic activity by about 1.7-fold and 30 *μ*M by 6-fold (Figure 1(b), neuron monoculture). Astrocytes appeared more resistant to oxaliplatin toxicity since cell viability was decreased by about 40% and 55% after treatment with 10 *μ*M and 100 *μ*M oxaliplatin, respectively (Figure 1(a), astrocyte monoculture). One *μ*M oxaliplatin (48 h incubation) was unable to modify both cell viability (Figure 1(b), astrocyte monoculture) and apoptotic processes (103.4 ± 12.5% versus control 100.0 ± 0.13%).

**Figure 2:**
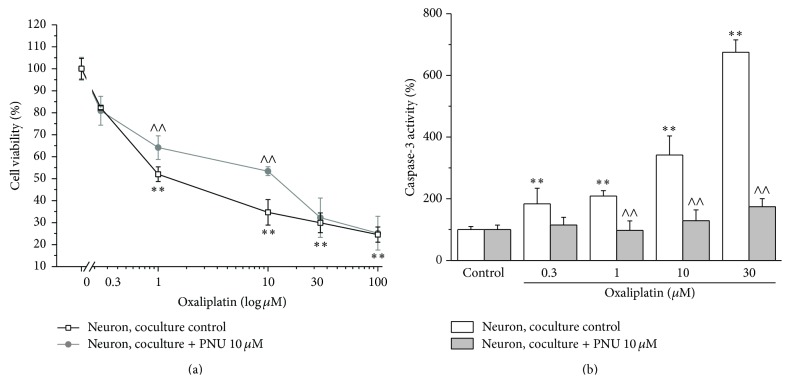
Effect of PNU in cocultures. (a) Cell viability. Neuron astrocyte coculture (2 10^5^ neurons/well and 8 10^4^ astrocytes/well) was incubated with oxaliplatin (0.3–100 *μ*M) in the absence or presence of 10 *μ*M PNU for 48 h. Neuronal cell viability was quantified by MTT assay. Values are expressed in percentage of control absorbance as mean ± S.E.M. of 6 experiments. Control condition absorbance was fixed to 100%. ^*∗∗*^ *P* < 0.01 versus control; ^∧∧^ *P* < 0.01 versus oxaliplatin treatment. (b) Caspase-3 activity. Neuron astrocyte coculture (7 10^5^ neurons/well and 4.5 10^5^ astrocytes/well) was incubated with oxaliplatin (0.3–100 *μ*M) in the absence or presence of 10 *μ*M PNU for 48 h. Neuronal enzymatic activity was measured by a fluorescent assay. Values are expressed as percent of control caspase-3 activity arbitrarily set as 100%. Bars represent mean ± S.E.M. of 3 experiments. One-way ANOVA was performed followed by a Bonferroni significant difference procedure. ^*∗∗*^ *P* < 0.01 versus control; ^∧∧^ *P* < 0.01 versus oxaliplatin treatment.

**Figure 3:**
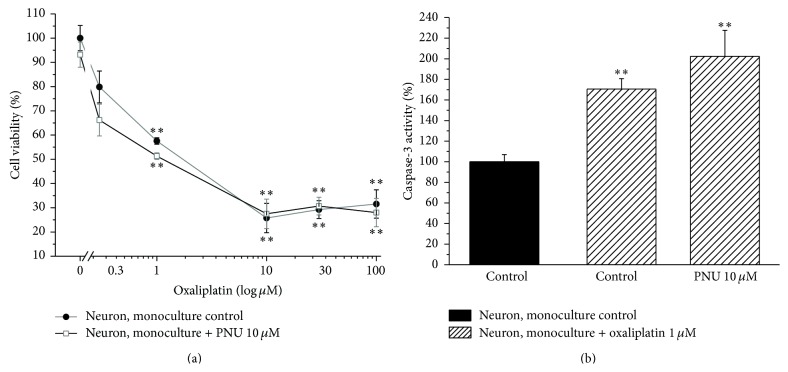
Effect of PNU in neuron monoculture. (a) Cell viability. Neurons (10^4^ cells/well) were incubated with oxaliplatin (0.3–100 *μ*M) in the absence or presence of 10 *μ*M PNU for 48 h. Viability was quantified by MTT assay. Values are expressed in percentage of control absorbance as mean ± S.E.M. of 6 experiments. Control condition absorbance was fixed to 100%. ^*∗∗*^ *P* < 0.01 versus control. (b) Caspase-3 activity. Neurons (7 10^5^ cells/well) were incubated with 1 *μ*M oxaliplatin in the absence or presence of 10 *μ*M PNU for 48 h. The enzymatic activity was measured by a fluorescent assay. Values are expressed as percent of control caspase-3 activity arbitrarily set as 100%. One-way ANOVA was performed followed by a Bonferroni significant difference procedure. Bars represent mean ± S.E.M. of 3 experiments. ^*∗∗*^ *P* < 0.01 versus control.

Figure 1 (neuron coculture) shows the response of neurons to oxaliplatin treatment when cocultured with astrocytes. As compared to neuron monoculture, the presence of astrocytes did not significantly modify oxaliplatin toxicity evaluated as cell viability as well as caspase-3 activity. When neuron astrocyte cocultures were incubated with the *α*7 nAChR agonist PNU (10 *μ*M), the neuronal damage was strongly reduced (Figure 2). As shown, PNU increased neuron viability by 10% and 20% in cocultures treated with 1 and 10 *μ*M oxaliplatin, respectively (Figure 2(a)). Moreover, PNU completely prevented the activation of caspase-3 up to 30 *μ*M oxaliplatin (Figure 2(b)). In the absence of astrocytes, PNU did not protect neurons from oxaliplatin neurotoxicity since cell viability of neuron monoculture was reduced by 40% after 48 h incubation with 1 *μ*M oxaliplatin and by 70% for the concentration range 10–100 *μ*M (Figure 3(a)). Similarly, 10 *μ*M PNU did not decrease caspase-3 activity induced by 48 h incubation with 1 *μ*M oxaliplatin (Figure 3(b)).

**Figure 4:**
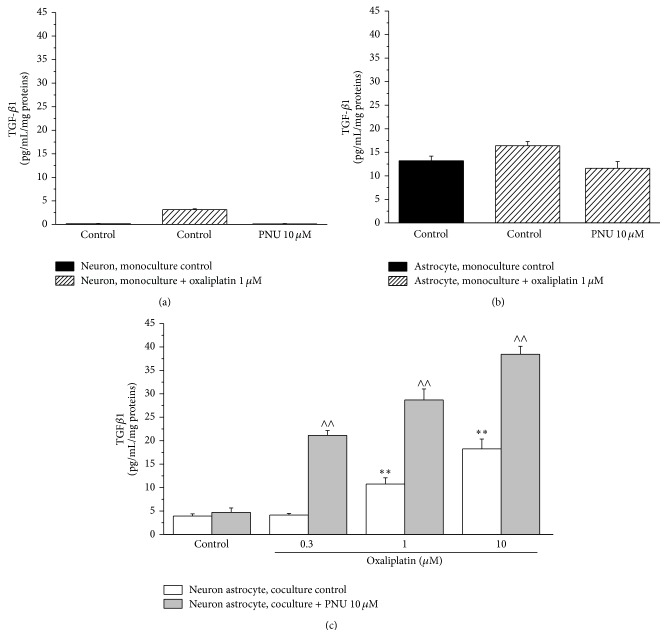
TGF-*β*1 dosage. The concentration of the anti-inflammatory cytokine TGF-*β*1 was measured in the culture medium of (a) neuron, (b) astrocyte monoculture, and (c) neuron astrocyte coculture. ((a) and (b)) Cells were treated with 1 *μ*M oxaliplatin (48 h) in the absence or presence of 10 *μ*M PNU. (c) Cocultures were treated with increasing concentrations of oxaliplatin (0.3, 1, and 10 *μ*M) in the absence or presence of 10 *μ*M PNU. Results are expressed as pg/mL of TGF-*β*1 per mg of proteins. Values are expressed as the mean ± S.E.M. of 6 experiments. One-way ANOVA was performed followed by a Bonferroni significant difference procedure. ^*∗∗*^ *P* < 0.01 versus control; ^∧∧^ *P* < 0.01 versus oxaliplatin treatment.

Figure 4 shows TGF-*β*1 concentrations in culture media after 48 h incubation (oxaliplatin 1 *μ*M). The basal production of TGF-*β*1 was 0.14 ± 0.05 pg/mL in neuron monoculture and 13.2 ± 0.99 pg/mL in astrocytes. Neither oxaliplatin nor PNU significantly altered these levels. In the medium of neuron astrocyte coculture, 3.93 ± 0.45 pg/mL was basally detected (48 h). Oxaliplatin (1 and 10 *μ*M) increased TGF-*β*1 release by about 2- and 3-fold with respect to control values. PNU (10 *μ*M) potentiated the stimulatory effect of oxaliplatin increasing the cytokine levels by 4-, 6-, and 8-fold in the presence of oxaliplatin of 0.3, 1, and 10 *μ*M (Figure 4).

In Table 1, the intracellular concentrations of pyruvate in astrocytes are shown. Oxaliplatin (1 *μ*M, 48 h) increased pyruvate by about 5-fold; this effect was not altered in the presence of 10 *μ*M PNU. PNU* per se* was not able to significantly modify basal pyruvate levels.

**Figure 5:**
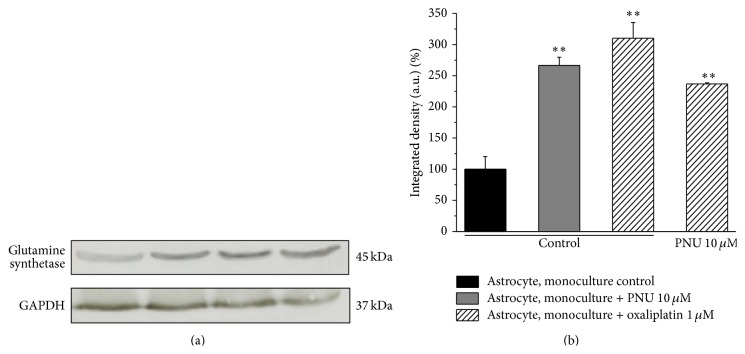
Glutamine synthetase expression level in astrocyte monoculture. Astrocytes were separately treated with 1 *μ*M oxaliplatin, 10 *μ*M PNU or coincubated with oxaliplatin and PNU for 48 h. Densitometric analysis (b) and representative western blot (a) are shown. GAPDH normalization was performed for each sample. Values are expressed as the mean ± S.E.M. of 3 experiments. One-way ANOVA was performed followed by a Bonferroni significant difference procedure. ^*∗∗*^ *P* < 0.01 versus control.

Glutamine synthetase expression levels were evaluated in astrocytes by western blot (Figure 5). After 48 h, oxaliplatin (1 *μ*M) and PNU (10 *μ*M) increased the enzyme expression by 2.5- and 3-fold, respectively. The concomitant treatment with oxaliplatin and PNU induced a 2.4-fold increase in glutamine synthetase expression (Figure 5).

## 4. Discussion

The present results highlight the neuronal protective effect induced by the *α*7 nAChR stimulation. The presence of astrocytes is needful to evoke the protective effect of PNU against the oxaliplatin neurotoxicity. In a rat model of oxaliplatin neuropathy (2.4 mg kg^−1^ intraperitoneally, daily for 21 days, according to [26]), we have previously described a painful condition characterized by neuronal damage associated with the activation of glial cells (in particular astrocytes) in spinal cord and in the “pain matrix” areas [11]. In this condition, the inorganic platinum plasmatic concentration is about 18 *μ*M [27]. Assuming the integrity of the blood-brain barrier, oxaliplatin has about 5% distribution in the central nervous system (CNS) [28, 29] allowing hypothesizing a concentration near to 1 *μ*M. To note, the animal experimental protocol is consistent with the clinical use of the antitumor drug [27]. In the present cell culture experiments, neurons are more prone than glia to oxaliplatin damage since both cell mortality and apoptosis are induced by lower concentrations in neurons than in astrocytes. In particular, the incubation for 48 h with 1 *μ*M oxaliplatin (a concentration in agreement with the CNS theoretical one of* in vivo* experiments) impairs neurons without negatively affecting glia.

Astrocytes are generally less susceptible to injuries than neurons and can exert neuroprotective effects [13, 30]; on the other hand, astroglia is derived from a neuroectoderm lineage and supports the maintenance of central nervous system homeostasis [31]. Astrocyte activation can protect neurons by preserving bioenergetics [32], providing a trophic support [33], preventing excitatory neurotoxicity [34, 35], and modulating free radical oxidation [36, 37] and apoptosis [38] in neurons. Unfortunately, in neuropathic conditions dysfunctional glial cells no longer maintain homeostasis and even contribute to nervous circuit alterations [12, 39]. In the present study, oxaliplatin increases pyruvate intracellular concentration of astrocytes and this increase might be an attempt to counterbalance oxaliplatin toxicity. In astrocytes, pyruvate derived from glucose may be oxidized in mitochondria to produce ATP by the tricarboxylic acid cycle [40]. Therefore, an increase in intracellular pyruvate suggests the activation of the energy metabolism. Nevertheless, astrocytes* per se* are not able to prevent the neuronal damage induced by oxaliplatin and the *α*7 nAChR stimulation is needed to improve the astrocyte protective role.

The neuroprotective role of *α*7 nAChR has been described* in vitro* [41] and* in vivo* in both the peripheral [18, 42] and central nervous system [43]. *α*7 nAChR-dependent activation of glial cells is observed [17] and *α*7 nAChR agonists are able to preserve nervous tissue in neuropathic states [17, 42] as well as in neurodegenerative conditions such as Parkinson's and Alzheimer's diseases [43]. In neurons, survival signal transduction by the phosphatidylinositol 3-kinase/AKT pathway (PI3K/AKT), the Janus kinase-2/signal transducer and activator of transcription-3 (JAK2/STAT3) pathway, and the mitogen activated protein kinase/extracellular signal regulated kinase (MAPK/ERK) pathway participate in *α*7 nAChR-mediated neuroprotection [44, 45]. Activation of microglial *α*7 nAChR during neuroinflammation modulates inflammatory cytokine (like TNF-*α*) release with protective effects on neurons [46] and the *α*7 nAChR-dependent Ca^2+^ influx into microglia may stimulate *β*-amyloid phagocytosis through actin reorganization [45]. As regards astrocytes, the *α*7 nAChR activation is able to inhibit TNF-*α* release and MAPK in astrocytes activated by 1-methyl-4-phenylpyridinium ion (MPP+) or lipopolysaccharide [47]. Moreover, *α*7 nAChR is protective for astrocytes since, through the stimulation of *α*7 nAChR, nicotine suppresses astrocyte apoptosis induced by oxidative stress [47]. Nevertheless, insufficient information exists about the positive role that the *α*7 nAChR stimulation mediates in astrocyte neuroprotective signaling. The present results show that the *α*7 nAChR agonist PNU is able to rescue neurons from oxaliplatin toxicity only in the presence of astrocyte cells highlighting the central role of astroglia in the protective signals evoked by *α*7 nAChR. Neuron survival and inhibition of apoptotic processes are correlated with enhanced release of TGF-*β*1 in coculture. The basal level of the growth factor is higher in astrocytes than in neurons but in monoculture neither oxaliplatin nor PNU is able to evoke a cytokine modulation. Differently, in coculture TGF-*β*1 increases up to 8-fold in the presence of PNU and, at a lesser extent, by oxaliplatin* per se*. TGF-*β*1 is a member of the TGF-*β* superfamily and it has been implicated in such diverse processes as regulation of growth, differentiation, extracellular matrix formation, and immune regulation as well as induction of neuronal survival and repair following injury [48, 49]. TGF-*β*1 is recognized as determinant in the protective astrocytic functions for neuron homeostasis by the c-Jun/AP-1 transcription factor pathway [50]. The role of TGF-*β*1 in the orchestration of repair processes has been evidenced in many neurodegenerative diseases and following vascular accident in the brain [51–53]; furthermore, TGF-*β*1 attenuates spinal neuroinflammation and excitatory amino acid system in rats with neuropathic pain [54], leading to pain relief after intrathecal administration [55]. TGF-*β*1 inhibits the production and release of proinflammatory cytokines, nitric oxide, and oxygen free radicals [56, 57]. In addition, TGF-*β*1 modulates the high synaptic glutamate levels associated with neurotoxic and neurodegenerative processes [58], increasing the expression of the glutamate-aspartate transporter (GLAST [59]) expressed preferentially in astrocytes [60]. Besides glutamate transporters, the glutamate-glutamine metabolic cycle between astrocytes and neurons is believed to be vital for preventing neuronal excitotoxicity [61]. In the astrocytic intracellular milieu glutamate is rapidly converted to glutamine by glutamine synthetase [62], an astrocyte-specific enzyme [63]. Glutamine can be shuttled out of glial cells and taken up by neurons for use by glutaminase in the glutamine cycle [64]. The coupling of glutamine synthetase and glutamine traffic from glia to neurons permits glutamate passage in the extracellular compartment in a nonneuroactive form (glutamine) thus avoiding toxicity [65]. Recently, Zou and coworkers [66] have demonstrated that the downregulation of glutamine synthetase reduces astrocyte protection against glutamate excitotoxicity to neurons. In the present study, glutamine synthetase expression is significantly upregulated in astrocyte cells after oxaliplatin treatment, suggesting that glia could preserve neurons from glutamate-induced excitotoxicity through this mechanism. This protective response is promoted by cell incubation with PNU alone and maintained after the cotreatment with oxaliplatin + PNU. On the other hand, glutamine synthetase may be stimulated by the increased level of pyruvate. Pyruvate is substrate of pyruvate carboxylase and pyruvate dehydrogenase forming the tricarboxylic acid cycle constituent citrate, from which glutamate is generated via *α*-ketoglutarate [67]. PNU* per se* does not alter pyruvate level; therefore, astrocytes stimulated by this *α*7 nAChR agonist might optimize their protective role without favoring glutamate toxicity.

## 5. Conclusions

These results are a contribution to the comprehension of the neuroprotective signaling evoked by the *α*7 nAChR stimulation. The positive role of astrocyte activation is highlighted concomitantly with the increase of TGF-*β*1 and glutamine synthetase, two biological mediators of neuroprotection also able to mediate pain relief. The *α*7 nAChR modulation of the pathological neuron-glia cross talk could control oxaliplatin neurotoxicity.

## Figures and Tables

**Table 1:** Astrocyte monoculture. Pyruvate intracellular levels.

Pyruvate (%)			
Control	Oxaliplatin (1 *μ*M)	PNU (10 *μ*M)	Oxaliplatin (1 *μ*M) + PNU (10 *μ*M)
100.0 ± 16.3	545.9 ± 56.7^*∗∗*^	129.8 ± 25.3	496.5 ± 38.2^*∗∗*^

Pyruvate intracellular concentration was measured in astrocyte monoculture. Cells were treated with 1 *μ*M oxaliplatin (48 h) in the absence or presence of 10 *μ*M PNU. Values are expressed in percentage of control as mean ± S.E.M. of 6 experiments. Control condition was fixed to 100%. One-way ANOVA was performed followed by a Bonferroni significant difference procedure. ^*∗∗*^ *P* < 0.01 versus control.
